# Neuromodulation and Behavioral Flexibility in Larval Zebrafish: From Neurotransmitters to Circuits

**DOI:** 10.3389/fnmol.2021.718951

**Published:** 2021-07-15

**Authors:** Laura Corradi, Alessandro Filosa

**Affiliations:** Max-Delbrück-Center for Molecular Medicine in the Helmholtz Association (MDC), Berlin, Germany

**Keywords:** zebrafish, neuromodulation, behavior, neuronal circuits, flexibility, foraging, escape (avoidance), arousal

## Abstract

Animals adapt their behaviors to their ever-changing needs. Internal states, such as hunger, fear, stress, and arousal are important behavioral modulators controlling the way an organism perceives sensory stimuli and reacts to them. The translucent zebrafish larva is an ideal model organism for studying neuronal circuits regulating brain states, owning to the possibility of easy imaging and manipulating activity of genetically identified neurons while the animal performs stereotyped and well-characterized behaviors. The main neuromodulatory circuits present in mammals can also be found in the larval zebrafish brain, with the advantage that they contain small numbers of neurons. Importantly, imaging and behavioral techniques can be combined with methods for generating targeted genetic modifications to reveal the molecular underpinnings mediating the functions of such circuits. In this review we discuss how studying the larval zebrafish brain has contributed to advance our understanding of circuits and molecular mechanisms regulating neuromodulation and behavioral flexibility.

## Introduction

Animals live in dynamic environments, wherein to survive and thrive they need to adapt their behaviors on the basis of information from the external world acquired through sensory systems, and internal states coding for their physiological needs. For example, foraging behavior is regulated by internal factors such as hunger, and external variables including the availability of food and presence of potential threats (Lima and Dill, 1990; Mobbs et al., 2018). Studies conducted in invertebrates and vertebrates have identified several neuromodulatory circuits and molecular mechanisms responsible for regulating the way an animal integrates internal states and external stimuli to make flexible behavioral adjustments (Bargmann, 2012; Lee and Dan, 2012; Marder, 2012; Kennedy et al., 2014; Kim S. M. et al., 2017).

Here we will summarize recent discoveries that are giving us fresh insights into the mechanisms mediating neuromodulation in zebrafish larvae. The young zebrafish brain is a useful model system to identify neuronal circuits mediating neuromodulation and to characterize the effects of manipulations of their activity on downstream neurons and consequent behavioral alterations. Transgenesis methods allow the generation of fish lines labeling specific populations of neuromodulatory neurons (Kawakami et al., 2010; Förster et al., 2017). Optogenetic, chemogenetic, and targeted mutagenesis techniques allow altering their activity to test their role in sensory processing and generation of motor outputs (Curado et al., 2008; Baier and Scott, 2009; Friedrich et al., 2010; Wyart and Del Bene, 2011; Li et al., 2016; Dal Maschio et al., 2017; Vanwalleghem et al., 2018). Moreover, methods for imaging and analyzing activity of a large number of neurons are lowering the barrier for discovering new neuromodulatory circuits (Ahrens et al., 2012; Freeman et al., 2014; Keller and Ahrens, 2015; Randlett et al., 2015; Lovett-Barron et al., 2017; Mu et al., 2020). Finally, zebrafish larvae perform several well-characterized behaviors (Budick and O’Malley, 2000; Orger and de Polavieja, 2017), some of which, such as hunting and threat avoidance, are modulated by internal states (De Marco et al., 2014, 2016; Filosa et al., 2016; Lovett-Barron et al., 2017). The behavioral repertoire of older zebrafish is wider, including for example territoriality, social, and reproductive behavior (Orger and de Polavieja, 2017), but their larger and optically-opaque brains constitute an obstacle for optical interrogation of neuronal circuits involved in neuromodulation. We will review mostly research performed in up to 2 weeks old larvae, when the applicability of powerful methods for optical interrogation of circuits is optimal. We will also discuss some findings related to learning in juvenile (up to 4 weeks old) zebrafish.

The large majority of neuromodulatory systems present in mammals can also be found in larval zebrafish, with the advantage of being composed of small amounts of neurons (often less than one hundred). A comprehensive description of neuromodulatory circuits present in zebrafish is beyond the scope of this review, and we suggest the interested reader to consult very detailed studies published before (Kaslin and Panula, 2001; Rink and Wullimann, 2002; McLean and Fetcho, 2004; Kastenhuber et al., 2010; Filippi et al., 2010; Panula et al., 2010; Tay et al., 2011; Semenova et al., 2014; Herget and Ryu, 2015). We will instead introduce several examples of neuromodulators in relation to their actions on specific neuronal circuits and behaviors. Since the main tasks of a young zebrafish are searching for food and avoid becoming food for predators, this review will mainly focus on mechanisms modulating foraging and defensive behaviors. We will also discuss the regulation of arousal states, which have profound influences on sensory processing and execution of motor actions.

## Modulation of Exploratory and Foraging Behaviors

Animals need to explore their environment to find food, employing foraging strategies aimed at maximizing food procurement and minimizing energy expenditure and risk of encountering predators (Lima and Dill, 1990; Mobbs et al., 2018). Zebrafish larvae start navigating their surroundings immediately after hatching, using a locomotion pattern characterized by alternating rapid bursts of swimming and short intervals of inactivity (Drapeau et al., 2002). Neurons of the reticulospinal network residing in the brainstem, the nucleus of the medial longitudinal fasciculus (nMLF) in the midbrain, and downstream circuits in the spinal cord are responsible for regulating locomotion (Orger et al., 2008; Fetcho and Mclean, 2010; Severi et al., 2014; Berg et al., 2018). Brain and spinal motor circuits are under the influence of neuromodulators. For example, the dopaminergic system is an important player in the motor control of vertebrates (Grillner and Robertson, 2016), including zebrafish larvae (Lambert et al., 2012; McPherson et al., 2016; Barrios et al., 2020). Both in mammals and teleost fish, dopaminergic neurons can be identified by the expression of the gene *tyrosine hydroxylase* (*th*), encoding one of the enzymes necessary for dopamine synthesis. Zebrafish has two paralogous *th* genes (*th1* and *th2*) displaying partially non-overlapping expression patterns (Filippi et al., 2010). The activity of subpopulations of hypothalamic th1^+^ and th2^+^ dopaminergic neurons is correlated with swimming (Jay et al., 2015; Reinig et al., 2017; Barrios et al., 2020). Ablation of hypothalamic dopaminergic neurons decreased the frequency of swimming bouts (McPherson et al., 2016; Barrios et al., 2020). Consistently, optogenetic activation of th2^+^ cells increased locomotor activity (McPherson et al., 2016; Barrios et al., 2020). Together, these data suggest that hypothalamic dopaminergic neurons modulate locomotion, and may be part of a neuronal circuit controlling exploratory behavior. It would be interesting to know if and how motivational systems controlling energy balance communicate with these dopaminergic neurons to regulate foraging-related exploration in response to internal homeostatic needs.

**Figure 1:**
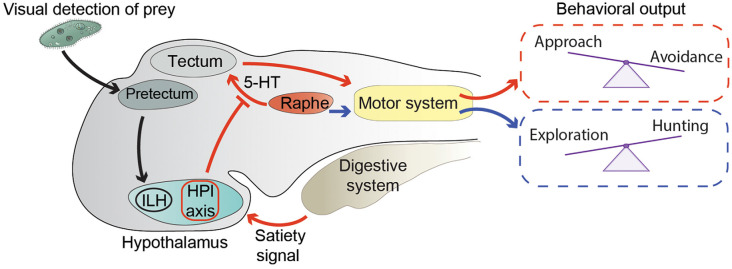
Neuronal circuits modulating exploration and hunting behavior. Black arrows represent the circuit conveying visual information about prey from the pretectum to the inferior lobe of the hypothalamus (ILH; Muto et al., 2017). Red arrows summarize the way information about energy status modulates the hunting/escape decision (Filosa et al., 2016). An unidentified satiety signal activates the hypothalamus-pituitary-interrenal (HPI) axis. In addition, satiety leads to inhibition of serotonergic (5-HT) neurons in the dorsal raphe nucleus, possibly through the action of the HPI axis. Activation of the HPI axis and inhibition of the dorsal raphe lead to altered processing in the tectum of visual stimuli representing preys or potential threats. Changes in visual processing are then likely translated into motor commands controlling the selection of motor actions required to either approach a prey or escape from a threat. Blue arrows represent the correlation between hunting behavior and activity of 5-HT neurons of the dorsal raphe, and their possible involvement in the transition from exploration to hunting behavior (Marques et al., 2020).

The main function of exploratory behavior in a young zebrafish is to find food. When a zebrafish larva encounters a potentially edible object, it needs to switch its behavior from exploration, when it travels relatively large distances, to prey hunting in its immediate surroundings. Larvae hunt preys (in laboratory settings most often unicellular organisms such as paramecia, or small moving visual stimuli simulating them) with a well-coordinated series of fin, tail, eye, and jaw movements performed in sequential steps of prey detection, approach, and ingestion (Budick and O’Malley, 2000; Bianco et al., 2011; Preuss et al., 2014; Semmelhack et al., 2014; Antinucci et al., 2019; Mearns et al., 2020). By using a microscope capable of performing whole-brain calcium imaging at cellular resolution in freely swimming zebrafish larvae (Kim D. H. et al., 2017), Marques et al. identified neuronal correlates of exploration and hunting behavior (Marques et al., 2020). Notably, serotonergic neurons in the dorsal raphe nucleus were active during hunting, suggesting that they may be involved in triggering the transition from exploration to hunting behavior (Figure 1). This hypothesis is in agreement with a reduction of hunting attempts made by larvae lacking a functional serotonergic system (Filosa et al., 2016). In the same study, Marques et al. revealed correlations of activity of other neuromodulatory populations of neurons with specific steps of the hunting sequence. For example, tectal cholinergic neurons were active during prey detection. Other neurons including dopaminergic ones in the hindbrain, noradrenergic cells of the locus coeruleus, and cholinergic neurons in the cerebellum were preferentially activated by hunting success, possibly suggesting their involvement in reward systems. It would be worthwhile to follow up this study with interventional methods (optogenetic manipulation or neuronal ablations) to test a causative link between the activity of different types of neuromodulatory neurons and subcomponents of hunting behavior.

The dorsal raphe nucleus was also found to be involved in the decision process of approaching or avoiding visual stimuli that could possibly represent food (Filosa et al., 2016). Foraging animals search for food, and at the same time must try not to become food for predators by employing effective threat-avoidance strategies (Lima and Dill, 1990). In zebrafish larvae, the choice between prey-approach and threat-avoidance behavior is strongly influenced by their feeding state (Figure 1). When confronted with ambiguous visual stimuli that could either indicate the presence of food or a potential threat, satiated larvae tend to avoid them, displaying a loss-aversion strategy, while food-deprived fish are more prone to approach them, suggesting that they adopt a more risk-taking approach (Filosa et al., 2016). At the circuit level, modulation of feeding state of the approach/avoidance decision is mediated by the serotonergic system and the hypothalamus-pituitary-interrenal (HPI) axis (homologous to the mammalian hypothalamus-pituitary-adrenal axis), a neuroendocrine hub regulating responses to stress (Löhr and Hammerschmidt, 2011). Indeed, food intake activated the HPI axis, and boosting activation of the HPI axis by mutating the gene encoding the transcription factor glucocorticoid receptor (Ziv et al., 2012) mimicked the effect of satiation on behavioral choice (Filosa et al., 2016). On the other hand, food deprivation led to increased activity of serotonergic neurons of the dorsal raphe nucleus, and pharmacological enhancement of serotonergic transmission with the selective serotonin reuptake inhibitor fluoxetine in satiated larvae induced them to behave like food-deprived ones. Ablation of the serotonergic system in food-deprived fish had the opposite effect on behavioral selection (Filosa et al., 2016). The serotonergic system and the HPI axis are able to influence the approach/avoidance decision at early stages of sensory-motor transformations (Figure 1). Indeed, they both alter the representation of small (prey-like) and large (predator-like) visual stimuli in the optic tectum, the major visual-processing structure in the zebrafish brain, involved in both prey capture and threat avoidance behaviors (Roeser and Baier, 2003; Del Bene et al., 2010; Nevin et al., 2010; Preuss et al., 2014; Barker and Baier, 2015; Temizer et al., 2015; Dunn et al., 2016; Bollmann, 2019). Food intake or activation of the HPI axis decreased, while pharmacological enhancement of serotonergic transmission increased, the number of tectal neurons responding to small visual stimuli (Filosa et al., 2016). In addition to the tectum, feeding-state might also modulate the activity of other visual processing and motor-initiating centers involved in prey capture and avoidance behavior. Further work will be required to test this hypothesis. It is also likely that other modulatory systems, apart from the HPI axis and the dorsal raphe nucleus, regulate sensory-motor transformation according to energy balance. At least some of these circuits probably reside in the hypothalamus, since it is a brain structure important for regulating food intake in zebrafish and other vertebrates (Löhr and Hammerschmidt, 2011; Sternson, 2013). In agreement with this hypothesis, it was shown that neurons in the inferior lobe of the hypothalamus (ILH) are activated once a paramecium appears in the visual field of a zebrafish larva (Muto et al., 2017; Figure 1). In the same study, prey-detecting pretectal neurons projecting axons to the ILH were identified, demonstrating the existence in zebrafish of a direct neuronal circuit linking a center processing prey-related visual cues, such as the pretectum (Semmelhack et al., 2014; Antinucci et al., 2019), with the hypothalamus. Neuronal circuits conveying information in the opposite direction, from the hypothalamus to visual-processing areas, also exist. A population of neurons in the rostral hypothalamus was shown to establish connections with the neuropil region of the tectum (Heap et al., 2017). These cells appear to inhibit tectal neurons, although it is not clear if through direct monosynaptic connections or via an indirect multisynaptic pathway. Taken together, these data suggest that, like in other vertebrates, in zebrafish larvae, hypothalamic circuits integrate sensory inputs with homeostatic information to fine-tune behavioral responses to external stimuli according to internal needs.

The circuits and molecular pathways sensing metabolic status and controlling food intake in zebrafish are likely very similar to the ones present in mammals. Several neuropeptides important for controlling food intake in mammals, such as agouti-related peptide (Agrp), neuropeptide Y (Npy), and hypocretin (Hcrt, also known as orexin) were shown to have conserved roles in zebrafish (Song and Cone, 2007; Yokobori et al., 2011, 2012). However, most of these studies were conducted in adult fish, and the neuronal circuits mediating the actions of the neuropeptides were not studied in detail. Few recent studies investigated how food intake and energy balance are regulated in larval zebrafish (Jordi et al., 2018; Shainer et al., 2019; Wee et al., 2019b). For example, two subpopulations of hypothalamic serotonergic neurons, differentially activated by prey detection and ingestion, were shown to be important to regulate food consumption (Wee et al., 2019b). More research at the circuit and molecular levels is required to have a complete picture of the hypothalamic networks regulating food intake in the larval zebrafish. Moreover, in order to have a better understanding of how hypothalamic circuits modulate behavioral flexibility, it is crucial to know how they interact with brain areas processing sensory information or generating motor commands. The optical accessibility and small size of the larval zebrafish hypothalamus are definitively great advantages for obtaining such information.

Learning and memory systems are crucial regulators of an animal’s behavior, enabling the comparison of current situations with stored information acquired in the past to avoid perpetuating erroneous actions and to improve motor outputs and behavioral choices through practice (Milner et al., 1998; McGaugh, 2000). Although some studies reported that zebrafish larvae appear to possess poor associative learning and memory capabilities (Valente et al., 2012; Yashina et al., 2019), several lines of evidence suggest that they are indeed able to learn associative tasks (Aizenberg and Schuman, 2011; Hinz et al., 2013; Lin et al., 2020). In accord with these latter reports, it was shown that learning is involved in prey-capture behavior performed by young fish. Larvae exposed to live prey (paramecia or rotifers) displayed elevated capture success compared to naïve larvae or controls raised with inert food (Lagogiannis et al., 2020; Oldfield et al., 2020). Moreover, compared to controls, prey-experienced fish had higher visually-evoked neuronal activity in the telencephalon and the habenula, suggesting that the two brain structures may contain neurons regulating learning processes required for refining motor sequences involved in hunting behavior (Oldfield et al., 2020). In support of this hypothesis, the ablation of habenular neurons led to a reduction of capture efficiency (Oldfield et al., 2020). More work is needed to identify the precise forebrain circuits responsible for this form of learning. Moreover, it would be interesting to know how neurons in the telencephalon and the habenula interact with sensory, motor, and homeostatic circuits to regulate behavioral performance.

## Control of Defensive Behaviors

Defensive behaviors are a class of innate responses fundamental for survival that involve detection of threatening stimuli and initiation of specific actions to avoid or eliminate the threat. Defensive strategies are influenced by external and internal factors, such as type and proximity of a threat and current metabolic needs, and elicit different behavioral responses including orienting the body away from the source of the hostile stimulus, freezing, hiding to avoid detection by predators, escaping to prevent capture, and suppressing foraging behavior to reduce risk of predation (Evans et al., 2019; Headley et al., 2019). These behaviors are associated with changes in the autonomic nervous system, which increases heart rate and blood pressure, as well as endocrine pathways mobilizing energy resources, that together allow proper responses to threats (Headley et al., 2019). Threat-avoidance behaviors require activation of sensory mechanisms that identify the type and intensity of the stimulus, hypothalamic circuits for both initiation of motor actions and activation of neuroendocrine responses, habenular circuits that promote aversive learning, and midbrain and hindbrain motor circuits that mediate fast escape responses.

Zebrafish has emerged as an excellent model organism for dissecting the neuronal circuits mediating defensive strategies. Indeed, stimuli that threaten fish homeostasis (e.g., exposure to hyperosmotic medium, temperature, and pH fluctuations), nociceptive stimuli (e.g., mustard oil, electric shock), and stimuli mimicking approaching predators (e.g., visual looming or acoustic/vibrational stimuli) are commonly used and well established (Burgess and Granato, 2007; Temizer et al., 2015; Dunn et al., 2016; Ryu and De Marco, 2017; Wee et al., 2019a; Yashina et al., 2019).

Detection of approaching predators in zebrafish larvae is mediated mainly by visual, somatosensory, and auditory systems (Korn and Faber, 2005; Medan and Preuss, 2014; Temizer et al., 2015; Dunn et al., 2016). Vibrational and acoustic stimuli activate hindbrain sensory circuits, which in turn relay information to a reticulospinal network of neurons, including the Mauthner cells, that trigger escape responses (O’Malley et al., 1996; Liu and Fetcho, 1999; Korn and Faber, 2005; Medan and Preuss, 2014). Looming visual stimuli activate neurons in the tectum and pretectum, and lesioning the tectum impairs efficient escape behavior (Temizer et al., 2015). Visual information about potential threats is also conveyed to reticulospinal escape circuits (Dunn et al., 2016).

**Figure 2:**
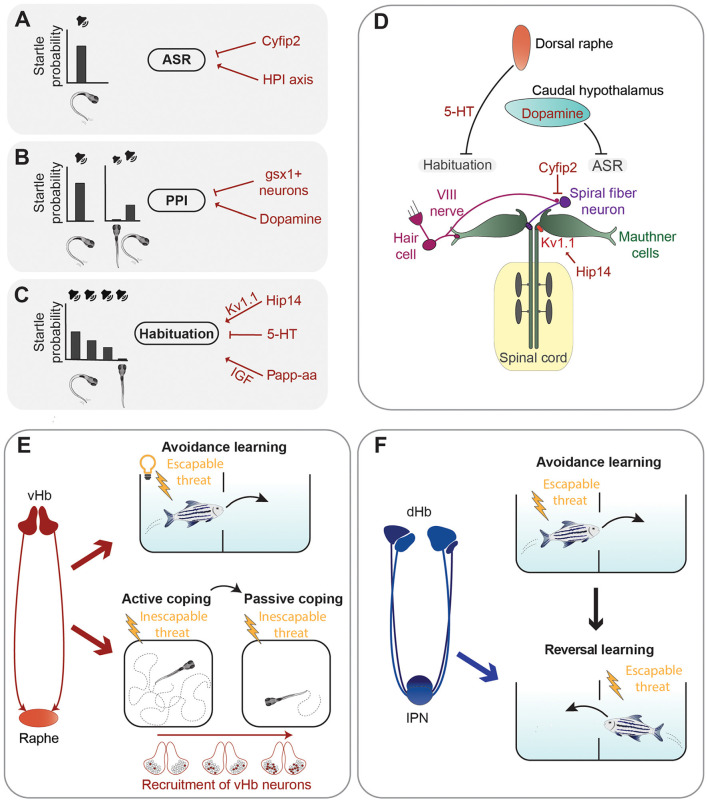
Summary of neuronal circuits and molecules regulating defensive behavior. **(A–C)** Left are representations of the acoustic stimuli and related probabilities to trigger an acoustic startle response (ASR). A loud sound efficiently triggers ASR **(A)**. When the acoustic stimulus is shortly preceded by a sub-threshold sound, the probability of triggering an ASR is reduced due to pre-pulse inhibition (PPI) **(B)**. Repetition of a supra-threshold sound with short intervals also leads to a reduction of the probability of triggering an ASR due to habituation **(C)**. Several neurotransmitters, proteins, and the HPI axis (in red) were found to control the execution of the ASR, PPI, or habituation **(A–C)**. **(D)** Graphical summary of the main circuits mediating the ASR and examples of neuromodulators regulating them. **(E)** The ventral habenula (vHb)–raphe network mediates avoidance learning in the presence of an aversive escapable stimulus (electric shock) and a conditioned stimulus (light; Amo et al., 2014). Sequential recruitment of vHb neurons controls the switch from active coping to passive coping behavior in the presence of an inescapable threat (electric shock; Andalman et al., 2019). **(F)** The dorsal habenula (dHb) is required for reversal learning (Palumbo et al., 2020), which involves updating previously acquired learning rules when new information is available (in this case change of the tank compartment associated with electric shock).

While escape responses need to be very fast to be effective, they can also be fine-tuned by neuromodulatory circuits. For example, the threshold for the stimulus intensity capable of eliciting an acoustic startle response (ASR), a fast unilateral C-bend of the tail which turns the animal away from the stimulus (Korn and Faber, 2005), and the speed of escape are not constant and can be regulated by several neuromodulators (Figures 2A–D). A forward genetic screen identified zebrafish mutants having increased startle sensitivity (Marsden et al., 2018). The authors mapped one of the mutations to the gene *cytoplasmic Fragile X mental retardation protein (FMRP)-interacting protein 2* (*cyfip2*), coding for a cytoskeleton-interacting protein which controls activity of excitatory spiral fiber neurons innervating the Mauthner cells (Marsden et al., 2018). The molecular mechanisms linking this protein regulating cytoskeletal functions to neuronal excitability are currently unknown. Another study showed that the serotonergic and dopaminergic systems regulate in opposite ways the speed of escape, with serotonergic agonists promoting slow escapes and dopaminergic receptor activators biasing behavior selection toward fast ones (Jain et al., 2018).

Escape behavior can be altered also by previous sensory experiences, such as in the case of pre-pulse inhibition (PPI) and habituation of the ASR. PPI is a sensorimotor gating phenomenon characterized by an attenuation of the startle response when it is preceded by a weak non-startling stimulus (Burgess and Granato, 2007; Figure 2B). The dopamine agonist apomorphine suppressed PPI of startle in larval zebrafish, while the dopamine D2 receptor antagonist haloperidol reduced the apomorphine effect, suggesting that the dopaminergic system inhibits PPI (Burgess and Granato, 2007). The exact populations of dopaminergic neurons responsible for this type of modulation have not been identified yet. A genetic screen identified hindbrain glutamatergic neurons, labeled by expression of the gene encoding the transcription factor Genomic Screen Homeobox 1 (*gsx1*), capable of promoting PPI (Bergeron et al., 2015). However, more work is required to have a complete picture of the neuronal circuits mediating PPI of startle responses in larval zebrafish.

Another way for previous sensory experience to modulate startle responses is through habituation, a form of non-associative learning, which leads to reduced response probability when the startling stimulus is presented repeatedly at short intervals (Wolman et al., 2011; López-Schier, 2019; Randlett et al., 2019; Figure 2C). Depression of dendritic excitability of the Mauthner cells is responsible for a short–term form of habituation of the ASR (Marsden and Granato, 2015). A forward genetic screen revealed some of the molecular mechanisms mediating habituation of the ASR by identifying the metalloprotease pregnancy-associated plasma protein-aa (Papp-aa) and the palmitoyltransferase Huntingtin interacting protein 14 (Hip14) as important regulators of this form of learning (Wolman et al., 2015; Nelson et al., 2020). Papp-aa modulates habituation by enhancing insulin-like growth factor (IGF) signaling (Wolman et al., 2015), while Hip14 regulates depression of sensory inputs onto dendrites of the Mauthner cells through palmitoylation of the Shaker-like K^+^ voltage-gated channel subunit Kv1.1 (Nelson et al., 2020). An interesting work showed that habituation of the ASR varies between different zebrafish larvae and that these inter-individual differences are stable over days and heritable (Pantoja et al., 2016). Notably, dorsal raphe serotonergic neurons, which project into the vicinity of the Mauthner cells and are also activated by acoustic stimuli, decrease their activity during habituation, and the amount of this decrease cosegregates with behavioral habituation between generations (Pantoja et al., 2016). These results suggest that inter-individual differences in the activity of dorsal raphe neurons might impact ASR habituation through the Mauthner cells network. Likely, other brain regions are involved in modulating inter-individual variability of escape responses. In a follow up study, Pantoja et al. (2020) raised two generations of fish selected for innate differences in the ASR habituation and mapped their whole-brain neuronal activity using immunostaining of phosphorylated extracellular signal-regulated kinase (pERK) as a readout of neuronal activation (Randlett et al., 2015). Larvae habituating faster to acoustic stimuli showed increased baseline activity in several brain regions including the caudal hypothalamus, which was previously reported to be involved in the modulation of ASR (Mu et al., 2012). In addition, fast-habituating individuals displayed a higher frequency of spontaneous activity in dopaminergic neurons of the caudal hypothalamus (Pantoja et al., 2020).

Neurons regulating arousal levels (discussed in the next section) and stress are also involved in modulating escape behavior in zebrafish. Indeed, it was shown that larvae with elevated activation of the HPI axis due to mutation of the glucocorticoid receptor (Ziv et al., 2012) display augmented ASR (Griffiths et al., 2012).

In fish, like in other vertebrates, the perception of threatening stimuli triggers the activation in the hypothalamus of the HPI axis, which leads ultimately to the synthesis and release of cortisol (Wendelaar Bonga, 1997; Alderman and Bernier, 2009). The key coordination center of the HPI axis is the neurosecretory preoptic region of the hypothalamus, analogous to the mammalian paraventricular nucleus (Herget and Ryu, 2015). Perception of stressors leads to activation in this region of corticotropin-releasing-hormone-producing (Crh^+^) neurons, which project directly to the pituitary and stimulate the corticotroph cells to release adrenocorticotropic hormone (Acth), which in turn triggers the secretion of cortisol from the interrenal gland (Ulrich-Lai and Herman, 2009; Löhr and Hammerschmidt, 2011). In support of the role of neuroendocrine systems in threat-evoked defensive behaviors, it was shown that enhancing the activity of pituitary corticotroph cells with optogenetic stimulation is sufficient to modulate locomotion and avoidance behavior immediately after the onset of a stressful stimulus (De Marco et al., 2016). Although endocrine responses are indubitably important to modulate behavior, several studies have revealed previously unknown roles for hypothalamic neurons in stress-related behaviors that are independent of hormonal actions (Fox and Lowry, 2013; Füzesi et al., 2016; Daviu et al., 2020). For example, Lovett-Barron et al. recently demonstrated that Crh^+^ neurons and oxytocin-producing neurons in the preoptic hypothalamus of zebrafish larvae can modulate fast behavioral responses to different homeostatic threats despite chemogenetic ablation of corticotroph cells in the anterior pituitary (Lovett-Barron et al., 2020). An interesting finding from this study is that different types of homeostatic threats—heat, salinity, acidity—and visual looming stimuli activate in the preoptic hypothalamus multiple populations of peptidergic neurons, rather than individual cell classes, which synergistically modulate rapid avoidance responses. This fast modulation of behavior might occur via glutamatergic synapses between the preoptic peptidergic neurons and brainstem spinal-projecting neurons (Lovett-Barron et al., 2020).

In addition to neuronal circuits promoting a certain motor action, systems must exist to suppress alternative conflicting behaviors happening at the same time (Kennedy et al., 2014). Circuits regulating stress may also work in this way. For example, it was shown that food intake is also affected by activation of the HPI axis (Carr, 2002; De Marco et al., 2014), as exposure to stressors, such as hyperosmotic medium or water motion, led to suppression of food consumption in zebrafish larvae (De Marco et al., 2014), possibly a mechanism to inhibit foraging behavior and let an animal concentrate on avoidance of potential threats.

Learning is another fundamental mechanism for survival as it confers behavioral flexibility for selecting threat-avoidance strategies. The habenula (Hb) has been implicated in the process of learning how to respond to aversive stimuli (Bianco and Wilson, 2009; Hu et al., 2020). In zebrafish, this structure is divided into dorsal habenula (dHb) and ventral habenula (vHb), which are homologous to the mammalian medial and lateral portions, respectively (Agetsuma et al., 2010; Amo et al., 2010). Zebrafish Hb receives inputs from the entopeduncular nucleus, hypothalamus, and median raphe (Beretta et al., 2012; Turner et al., 2016; Roberson and Halpern, 2018). While the dHb region projects to the interpeduncular nucleus (IPN), the vHb projects to the raphe (Agetsuma et al., 2010; Amo et al., 2010, 2014; Beretta et al., 2012; Roberson and Halpern, 2018). Most of the studies investigating the role of the habenula in zebrafish behavior have been performed with juvenile or adult fish.

Amo et al. (2014) demonstrated involvement of neurons in the vHb in active avoidance learning in adult zebrafish. The learning process investigated in this study was based on a conditioning protocol in which a light cue is presented as a conditioned stimulus to freely swimming fish, followed by an electrical shock. After several trials, fish eventually learned to associate the cue with the shock, the unconditioned stimulus, and to actively swim in another compartment of the tank to avoid the potential danger (Figure 2E). As the animal learned to associate the cue with the electric shock the authors observed, by using *in vivo* electrophysiology techniques, an increase of tonic activity of vHb neurons. Interestingly, optogenetic activation of vHb neurons led to increased activity of serotonergic neurons in the median raphe, which receives glutamatergic inputs from the vHb. Specific inactivation of the vHb-median raphe pathway abrogated the increase of tonic activity and impaired avoidance learning. Thus, tonic activity of vHb neurons, together with its projections to the median raphe, seems to be essential in encoding the reward expectation value of an aversive stimulus and thus induce escape from a potentially dangerous environment (Amo et al., 2014).

In the conditioning protocol used by Amo et al. (2014), fish are presented with an escapable harmful threat and learn to actively avoid it, using an active coping strategy. Most animals, when repeatedly exposed to inescapable aversive stimuli, eventually stop trying to avoid them and switch from active coping behavior to a passive one, usually characterized by reduced locomotion and a state that in mammals is often referred to as “helpless” (Maier and Seligman, 2016). A recent study revealed that in zebrafish the vHb is involved not only in the process of active coping in the presence of stressful conditions, as just described, but also in switching from active to passive coping (Figure 2E). Andalman et al. (2019) induced this behavioral transition in juvenile zebrafish using continuous inescapable mild electric shocks and analyzed whole-brain neuronal activity during the switch from active to passive coping behavior. Not only the vHb increased its activity after the inescapable aversive stimuli but, intriguingly, this effect was due to the sequential recruitment of habenular neurons during the aversive experience. During the transition from active to passive coping an overall decrease of neuronal activity in the raphe nucleus was observed. To prove a causal relationship between passive coping and the vHb-raphe circuit, the authors showed that optogenetic activation of vHb neurons or inhibition of serotonergic neurons of the dorsal raphe reduced swimming speed, consistent with a passive coping state. Moreover, stimulation of the vHb led to a significant increase of the number of inhibitory responses in the raphe suggesting a model wherein inescapable shock activates vHb neurons, causing inhibition of raphe neurons and passive coping behavior. In a recent study, Mu et al. (2019) discovered interactions between noradrenergic neurons and radial glia in the hindbrain that trigger passivity to prevent futile actions in response to unsuccessful behavior. It would be interesting to know whether habenular circuits and the hindbrain neuronal-glia network trigger passivity independently in response to different types of stimuli, or if they are both parts of a larger system regulating passivity regardless of behavioral context.

While the vHb is symmetric, the dHb shows a pronounced left-right asymmetry with differences in size, gene expression, neuronal populations, and innervation (Beretta et al., 2012; Turner et al., 2016; Roberson and Halpern, 2018). This neuroanatomical asymmetry correlates in young larvae with functional differences, as olfactory stimuli mostly activate the right Hb whereas the left Hb responds to changes in light (Dreosti et al., 2014). However, this asymmetry of sensory inputs disappears at later stages (Fore et al., 2020). Disruption of habenular asymmetry led to the elevation of cortisol levels and anxiety-like behavior in adult zebrafish (Facchin et al., 2015). The dHb has also been linked to defensive behaviors in zebrafish, as disruption of the dHb abrogated the execution of innate and experience-dependent fear responses (Agetsuma et al., 2010; Lee et al., 2010; Duboué et al., 2017).

A recent study in juvenile zebrafish used a conditioned place avoidance learning protocol similar to the one used by Amo et al. (2014) to demonstrate that the dorsolateral habenula (dlHb) is not important for conditioned avoidance learning, but instead mediates reversal learning, which involves the ability to use new information to modify associations learned in the past (Palumbo et al., 2020; Figure 2F). These results, together with another study showing that silencing the dlHb-IPN pathway impairs decision-making in a foraging task (Cherng et al., 2020), suggest that the dlHb in zebrafish is not only critically involved in aversive responses but is also an important regulator of behavioral flexibility.

In summary, the studies described in this section have shed light on the molecular and cellular identities of some of the major components of neuronal circuits essential for the flexibility of aversive behavior in zebrafish. The challenge for the near future, in addition to better characterize their molecular and functional properties, is to understand how each of these brain subcomponents communicates with each other in order to coordinate the selection of the most appropriate defensive strategy in response to external and internal conditions.

## Regulation of Arousal States

A critical prerequisite for an animal to hunt preys and defend itself from predators is to be alert in order to quickly process information from the environment and react accordingly. An animal’s arousal level can be operationally defined by the frequency and/or intensity of spontaneous locomotor activity and responsiveness to sensory stimuli: states of high arousal are associated with elevated locomotion and/or sensory sensitivity (Pfaff et al., 2008).

**Figure 3:**
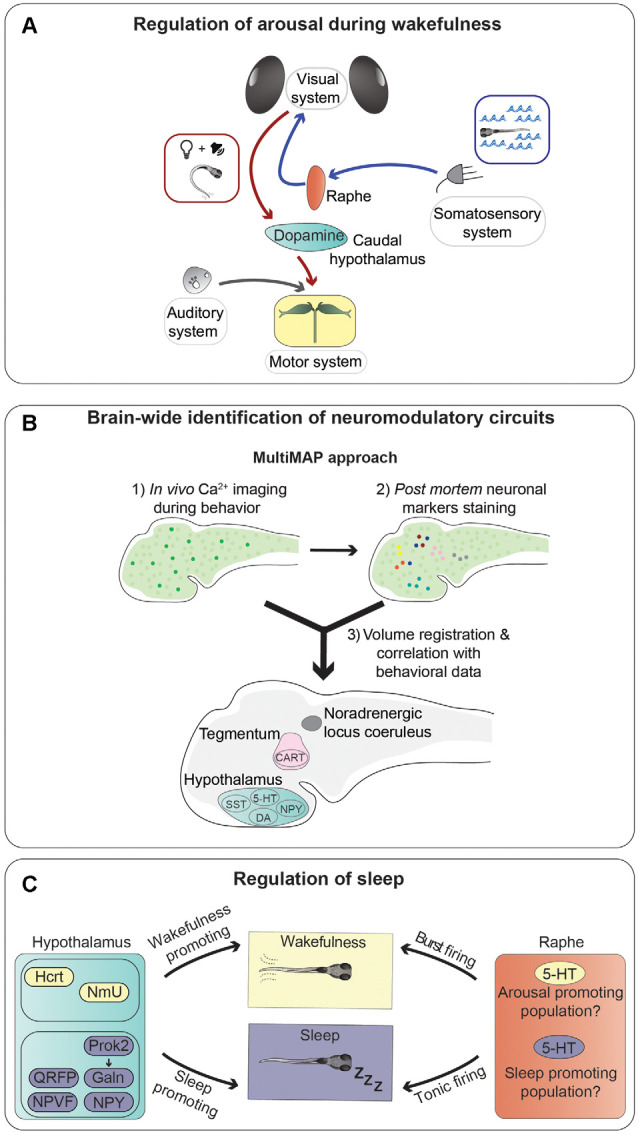
Neurotransmitters and neuronal populations controlling arousal and sleep. **(A)** Summary of the information flow involved in regulating trans-modal arousal. Water flow, through the somatosensory system, activates serotonergic neurons of the dorsal raphe nucleus, which in turn increase the sensitivity of the visual system to whole-field motion stimuli (blue arrows; Yokogawa et al., 2012). A flash of light preceding a sound facilitates the acoustic startle response by increasing responsiveness of the Mauthner cells, via activation of dopaminergic neurons in the hypothalamus (red arrows; Mu et al., 2012). **(B)** Novel whole-brain imaging techniques are facilitating the discovery of neuromodulatory circuits. This scheme summarizes a method (MultiMAP) combining *in vivo* calcium imaging with *post mortem* staining of neuronal markers to identify neurons whose activity correlates with specific brain states and/or behavioral tasks (Lovett-Barron et al., 2017). **(C)** Sleep is controlled by multiple brain regions and neurotransmitters. This graphical summary depicts the influences of several neuropeptides secreted by hypothalamic neurons (sleep-promoting in violet and wakefulness-promoting in yellow), and serotonergic cells of the raphe nucleus on sleep regulation. It was shown that the raphe can induce both arousal and sleep, possibly through different types of firing (tonic or burst), or maybe thanks to the presence of different populations of serotonergic neurons with specific connectivity patterns.

Several monoaminergic circuits play critical roles in regulating arousal in zebrafish. Among them is the noradrenergic system, as a homozygous mutation in the gene coding for the noradrenaline-synthesizing enzyme dopamine-beta-hydroxylase (*dbh*) leads to higher sensitivity to mechanoacoustic stimuli (Singh et al., 2015). Furthermore, serotonergic neurons of the dorsal raphe nucleus regulate arousal state and sensitivity to whole-field visual motion stimuli, which trigger the optomotor response (Yokogawa et al., 2012), a behavior involved in stabilization of body position in flowing water (Neuhauss et al., 1999). Indeed, it was shown that neuronal activity in the raphe increases when zebrafish larvae were exposed to sudden changes of water flow velocity, and ablation of serotonergic neurons in the raphe abolished the increase of visual sensitivity (Yokogawa et al., 2012; Figure 3A). Interestingly, water flow affected only the visual system, without altering other sensory modalities, suggesting the existence of neuronal mechanisms limiting the activation of sensory systems based on the relevant behavioral task. This is not an isolated case, since some neuropeptides were also found to have modality-specific arousal properties. For example, using an inducible overexpression method, Woods et al. showed that the neuropeptides Hcrt and Nociceptin modulate sensitivity to visual stimuli (Hcrt increases, while Nociceptin decreases responses to dark-flashes), but not to acoustic and thermal ones (Woods et al., 2014). This type of modality-selective arousal, a form of which was also observed in the mouse cerebral cortex (Shimaoka et al., 2018), is a very intriguing phenomenon that should be studied in more detail.

The regulation of visual sensitivity by a somatosensory stimulus mediated by the serotonergic system (Yokogawa et al., 2012) is not the only example of a cross-modal modulatory effect in zebrafish, as it was shown that a light flash stimulus is able to enhance sound-evoked escape behavior triggered by a subsequent auditory stimulus (Mu et al., 2012). The increase of escape probability was shown to be caused by light-responsive dopaminergic neurons residing in the caudal hypothalamus, which increased responsiveness of the Mauthner cells to auditory stimuli through activation of dopamine D1 receptors (Mu et al., 2012). The role of this type of cross-sensory modulatory effect is likely to increase the alertness of an animal following alterations of the visual environment, for example a flash of light that could signify the movement of a predator nearby, and react swiftly in response to further signals of potential danger, such as a suddenly appearing sound.

As mentioned earlier, one of the major advantages of using zebrafish larvae for studying neuromodulation is the fact that they have a small brain amenable to large-scale analysis of neuronal activity. Lovett-Barron et al. developed a semi-high-throughput technique, which they termed MultiMAP (Multiplexed alignment of Molecular and Activity Phenotypes), to identify molecularly-labeled populations of neurons whose activity correlates with specific behaviors (Lovett-Barron et al., 2017; Figure 3B). Using this method, the authors were able to identify several groups of neuromodulatory neurons regulating arousal in zebrafish larvae. They showed to the fish looming dark circles, simulating approaching predators, and measured reaction times of escape responses as a readout of alertness. Sensorimotor reaction times are inversely proportional to alertness: the higher the state of alertness, the faster the reaction time. First, calcium imaging was used to record the activity of large numbers of neurons right before the visual stimuli were presented, directly linking pre-stimulus neuronal activity to reaction times (arousal state). *Post mortem* immunofluorescence staining was then used to label several populations of neuromodulatory neurons. Volume registration of the calcium imaging and immunofluorescence data allowed to assign specific identities to neurons whose activity correlated with different arousal states. In this way, the authors were able to identify several groups of neurons active during high alertness states: cocaine- and amphetamine-regulated transcript (Cart) and cholinergic neurons in the tegmentum, noradrenergic cells of the locus coeruleus, and serotonergic, dopaminergic, and Npy-positive neurons in the hypothalamus. On the other hand, somatostatin-positive cells in the hypothalamus were correlated with low-alertness states. In the same study, several of the neuromodulatory systems identified in zebrafish were shown to have a conserved function also in mice. This work nicely shows the potential of using zebrafish larvae for the brain-wide discovery of evolutionarily conserved neuromodulatory networks.

The majority of animals cannot be on high alert for very long periods of time, and their arousal level needs to be tuned down in order to rest. Sleep is a resting behavioral state characterized by very low levels of arousal, in which thresholds for detection of sensory stimuli are lower compared to wakefulness. During sleep, behavioral activities including foraging and feeding are suppressed, while others, such as threat avoidance, can still be evoked but not as efficiently as during wakefulness. Sleep, or at least some form of rest, is present in a large variety of animals including mammals, birds, arthropods, worms, and even cnidarians (Campbell and Tobler, 1984; Anafi et al., 2019). Zebrafish larvae also display a clear alternation of high (diurnal) and low (nocturnal) motor activity during the day (Zhdanova et al., 2001; Chiu and Prober, 2013; Barlow and Rihel, 2017; Oikonomou and Prober, 2017). Zebrafish larvae exhibit the three criteria for sleep in non-mammalian animals (Campbell and Tobler, 1984; Anafi et al., 2019): a quiescent state under control of the circadian clock, increased arousal threshold (reduced responsiveness to sensory stimuli), and homeostatic regulation leading to sleep rebound after sleep deprivation. In some animals, such as mammals, birds, and reptiles, in addition to these behavioral manifestations, well-defined signatures of neuronal activity are also associated with sleep (Siegel, 2008; Shein-Idelson et al., 2016). However, until recently no data were reported about brain activity during sleep in zebrafish. By using whole-brain calcium imaging techniques, Leung et al. identified in the dorsal pallium of zebrafish neuronal signatures of sleep similar to those of slow-wave and rapid eye movement phases of mammals (Leung et al., 2019), suggesting that these patterns of neuronal activity might have evolved before the appearance of tetrapods.

Not surprisingly, there seems to be a functional overlap between some of the circuits modulating arousal during wakefulness and the ones regulating sleep. In some cases, they act in the same direction. For example, Hcrt increases arousal and decreases sleep. In other cases, the same brain structures and neurotransmitter systems can be involved in regulating both arousal and sleep in opposite directions. For instance, the serotonergic raphe nucleus was implicated in promoting arousal (Yokogawa et al., 2012), as mentioned earlier, but also in promoting initiation and maintenance of sleep (Oikonomou et al., 2019). Indeed, it was shown that serotonin receptor agonists or optogenetic stimulation of the raphe promote sleep, while pharmacological antagonization of serotonin signaling, inhibition of serotonin synthesis by mutating the gene coding for the serotonin-producing enzyme tryptophan hydroxylase 2 (*tph2*), or ablation of the raphe, reduce sleep. These seemingly contradictory results can be possibly explained by different patterns of neuronal firing required for the two functions. It was suggested that tonic activity of serotonergic neurons could have a sleep-promoting role, while burst firing would instead lead to increased arousal (Oikonomou et al., 2019; Figure 3C). In line with this hypothesis, in other species, distinct or even opposite consequences of tonic and burst firing have been reported for monoaminergic systems, including dopaminergic and noradrenergic ones (Aston-Jones and Cohen, 2005; Goto et al., 2007). Alternatively, or concomitantly, subpopulations of serotonergic neurons with different functions may exist within the raphe, as shown in mice (Okaty et al., 2015; Ren et al., 2018). It would be interesting to characterize, possibly in a systematic way, such diversity of subcomponents of neuromodulatory systems in zebrafish.

Another sleep-promoting molecule is melatonin produced by cells of the pineal gland (Kazimi and Cahill, 1999). Application of exogenous melatonin was shown to induce sleep in zebrafish (Zhdanova et al., 2001), while ablation of the pineal gland or mutating the gene coding for the melatonin-synthesizing enzyme (*aanat2*) caused a reduction of sleep (Gandhi et al., 2015). Molecular and behavioral circadian rhythms were not altered in *aanat2* homozygous mutants, suggesting that melatonin signaling is downstream of the circadian clock.

Neuropeptidergic signaling plays a major role in regulating sleep (Figure 3C). For example, the neuropeptide Hcrt has evolutionarily conserved functions in sleep regulation in both mammals and zebrafish (Sutcliffe and de Lecea, 2002; Prober et al., 2006). Overexpression of *hcrt* in zebrafish larvae has a wake-promoting effect (Prober et al., 2006), and chemogenetic activation of Hcrt-producing neurons reduces sleep (Chen S. et al., 2016). On the other hand, homozygous mutation of the *hypocretin receptor* (*hcrtr*) gene in adult zebrafish (Yokogawa et al., 2007), and ablation of Hcrt-producing neurons in larvae (Chen A. et al., 2016), caused sleep fragmentation.

One further neuropeptide involved in sleep regulation is Galanin (Galn), which is secreted by neurons located in the preoptic area and posterior region of the hypothalamus (Podlasz et al., 2012). The activity of Galn-positive neurons and levels of *galn* mRNA increase after administration of wake-promoting drugs or forced wakefulness (Reichert et al., 2019). Moreover, mutant fish larvae lacking Galn displayed a stark reduction of rebound sleep after sleep deprivation (Reichert et al., 2019). Together, these data suggest that this peptide may be involved in regulating the homeostatic component of sleep. However, Galn might be involved also in other aspects of sleep, since it was shown that it mediates the action of the neuropeptide Prokineticin 2 (Prok2) in promoting sleep in a light-dependent, but circadian-independent manner (Chen et al., 2017).

Several other neuropeptides were shown to control sleep. Among them, neuropeptide VF (NPVF; Lee et al., 2017), QRFP (Chen A. et al., 2016), and Npy (Singh et al., 2017) promote sleep, while Neuromedin U (NmU) is wake-promoting (Chiu et al., 2016). The presence of such a large variety of sleep-regulating neuropeptides suggests a complex role of neuropeptidergic transmission in inducing or inhibiting sleep. One of the factors contributing to this complexity is the existence of interactions between different populations of peptidergic neurons and signaling pathways and between neuropeptidergic and monoaminergic systems. While some of these interactions have been discovered, for example between Npy and noradrenergic neurons (Singh et al., 2017), NmU and Crh signaling (Chiu et al., 2016), or Hcrt-producing neurons with the melatonin-secreting pineal gland (Appelbaum et al., 2009) and with noradrenergic neurons of the locus coeruleus (Singh et al., 2015), a large amount of them very likely remains to be uncovered.

## Concluding Remarks and Future Directions

Innate behaviors are often thought as consisting of stereotypic and inflexible actions. However, this view has been challenged by studies demonstrating that even the most simple behaviors are flexible and can be altered by neuromodulatory systems in response to changes of external conditions or internal needs (Bargmann, 2012; Lee and Dan, 2012; Marder, 2012; Kennedy et al., 2014; Kim S. M. et al., 2017). A vast amount of this pioneering work was done in invertebrates. A current challenge is to apply the general principles of neuromodulation discovered in invertebrates to the specific functions of modulatory systems in vertebrates since neuronal circuits differ substantially between the two groups of animals. The zebrafish larva is an ideal model organism for bridging this gap, by combining some advantages of invertebrates, e.g., large numbers of progenies useful for screenings, and a small brain ideal for optical probing, with conserved anatomical and physiological properties in common with other vertebrates. In the past few years, important groundwork has been carried out to characterize the neuronal circuits mediating innate behaviors in zebrafish. Now is the time to investigate how neuromodulatory systems interact with these circuits to regulate behavioral flexibility.

The best-characterized circuit performing a sensorimotor transformation in zebrafish is the one responsible for the ASR. Therefore, it is not surprising that the first studies addressing the role of neuromodulation in zebrafish behavior focused on this particular circuit (Burgess and Granato, 2007; Wolman et al., 2011; López-Schier, 2019). This work showed that even a stereotypic and hardwired behavior such as the ASR is amenable to modification by several neuromodulators, and can be altered by non-associative forms of learning, such as habituation. The circuits responsible for more complex behaviors in zebrafish, such as hunting, are still not fully understood. However, brain regions and neuronal populations involved in hunting have been identified (Roeser and Baier, 2003; Semmelhack et al., 2014; Antinucci et al., 2019; Gebhardt et al., 2019; Förster et al., 2020), and recent work showed that at least some of them are influenced by neuromodulators (Filosa et al., 2016). Both hunting and threat avoidance are strongly influenced by arousal states, which regulate alertness levels and sensitivity to sensory stimuli, and can be regulated by learning and memory processes. It is still not clear how neuromodulatory processes, including arousal and learning, affect the full spectrum of behaviors in young zebrafish. Indeed, the behavioral repertoire of larval and juvenile zebrafish is not limited to hunting and threat avoidance. For example, they also display several aspects of social behavior (Dreosti et al., 2015; Hinz and de Polavieja, 2017; Larsch and Baier, 2018; Groneberg et al., 2020; Stednitz and Washbourne, 2020; Tunbak et al., 2020). However, the neuronal circuits mediating social behavior in zebrafish, and the modulatory systems regulating their activity, are still largely unknown. Moreover, we still don’t have a full catalog of all the molecules with neuromodulatory action, and complete knowledge of their functions is still lacking.

It is important to understand how neuromodulatory systems regulate the circuits controlling various types of behavior at different scales, from molecular alterations of signaling pathways to the activity of large ensembles of neurons. Strategies combining mutagenesis methods, behavioral screens, and whole-brain activity mapping in zebrafish larvae have the potential to reveal the whole picture of neuromodulation, from the micro to the macro scale, and to speed up the discovery of the mechanisms behind it. However, several challenges must be overcome before reaching a full understanding of the complexity of neuromodulatory systems. The first source of such intricacy is the large diversity of modulatory neurons and neurotransmitters. Often, the same neuronal circuit can influence multiple behaviors. This is the case, for example, of the serotonergic raphe nucleus, which in zebrafish larvae is involved in foraging (Marques et al., 2020), decision making during hunting (Filosa et al., 2016), regulation of visual sensitivity (Yokogawa et al., 2012), and sleep (Oikonomou et al., 2019). In other instances, multiple neuromodulatory systems are simultaneously engaged in regulating a single behavior, such as defensive responses to homeostatic threats (Lovett-Barron et al., 2020). A systems approach will be required to disentangle the intricate relationships between different types of neuromodulatory networks and the consequences of these interactions on different behaviors. The small and translucent brain of the young zebrafish will certainly have a central stage in this endeavor, also thanks to new techniques for whole-brain imaging of neuronal activity (Keller and Ahrens, 2015; Mu et al., 2020; Bruzzone et al., 2021), possibly in freely moving larvae (Kim D. H. et al., 2017), and novel behavioral tracking and classification methods (Marques et al., 2018). The second layer of complexity is presented by the fact that neuromodulatory neurons often project their axons to large areas of the brain and identifying their many downstream targets is challenging. Recent efforts to obtain information about the connectomes of large numbers of neurons in the central nervous system of zebrafish (Wanner et al., 2016; Hildebrand et al., 2017; Kunst et al., 2019) will certainly help in identifying connectivity patterns of neuromodulatory neurons. However, the application of these approaches to the study of neuromodulation is hindered by the fact that several neuromodulators can act far away from synapses or can be released at extrasynaptic sites (van den Pol, 2012). Novel strategies will likely be required to identify the neuronal targets of modulatory circuits in an efficient manner. Finally, the fact that multiple neuropeptides and non-protein neurotransmitters often coexist in the same neuron further complicates the study of neuromodulatory systems. Indeed, different neurotransmitters can be coreleased at the same synapse, or individually secreted from different axonal or dendritic locations (Ludwig and Leng, 2006; Vaaga et al., 2014; Nusbaum et al., 2017). Precise molecular manipulations for altering a single neurotransmitter signaling at a time in specific subgroups of neurons will be necessary to disentangle the functions of sympatric neuromodulators.

## Author Contributions

All authors contributed to the article and approved the submitted version.

## Conflict of Interest

The authors declare that the research was conducted in the absence of any commercial or financial relationships that could be construed as a potential conflict of interest.
